# Multi-indicator analysis of the influence of old municipal landfill sites on the aquatic environment: case study

**DOI:** 10.1007/s10661-019-7814-4

**Published:** 2019-11-26

**Authors:** Grzegorz Przydatek

**Affiliations:** Engineering Institute, State University of Applied Sciences in Nowy Sącz, Zamenhofa 1a street, 33-300, Nowy Sacz, Poland

**Keywords:** Indicators, Influence, Landfill, Waste, Aquatic environment, Leachate

## Abstract

The study aim was to analyse the influence of a municipal solid waste landfill site in operation for over 10 years on the aquatic environment using multiple indicators. The water around the landfill area must be controlled due to the possibility of leachate interaction with harmful substances in the environment. The tests were carried out on the basis of 24 indicators, of which four were the most significant: depth of groundwater retention, ammoniacal nitrogen (NH_4_-N), dissolved oxygen (DO), and polycyclic aromatic hydrocarbons (PAHs). The assessment of the quality of the surface water and groundwater and the analysis of the leachate pollution indices enabled the interpretation of the influence of a specific municipal waste landfill on the nearby water environment condition, despite not exceeding the permissible content at the highest average concentration of NH_4_-N at 1.34 mg L^−1^. The differences were significant at the level of *p* < 0.05 in the content of DO in the water below the landfill. The concentration of NH_4_-N in the groundwater below the landfill was statistically significantly correlated with the depth of the groundwater deposits (*r* = 0.609). Similarly, the surface water below the landfill site showed a statistically significant relation in the piezometer, which was also below the landfill, to ammoniacal nitrogen (*r* = 0.749). This result confirmed the statistically significant differences in the aquatic environment and the correlations with NH_4_-N and that, below the landfill, the penetration water seepage is moderate with a low waste accumulation not exceeding 10 Mg per day.

## Introduction

The increase in urbanisation and industrialisation affects the deterioration of surface water and groundwater quality (Su et al. 2011). One of the drivers of this increase is landfilling, which contributes to the pollution of drinking water resources (Kjeldsen et al. 2002; Lisk 1991). In addition, using landfills as a method of municipal solid waste management has a negative influence on water resources (Ramaroson et al. 2018). This form of waste management is still the most commonly used solution in the world (Laner et al. 2012). However, in some circumstances, the locations of landfills could be exposed to natural hazards, such as flooding and earthquakes (Hereher et al. 2019). On the other hand, Alslaibi et al. (2011) recognised the landfill as an engineered waste disposal site facility with specific pollution control technologies designed to minimise potential effects.

At the end of 2017, there were 301 landfills in Poland (Statistics Poland 2018). Due to the dominance of this form of waste management (Koda et al. 2017) in a country that is a member of the EU, special attention is paid to improving the management of municipal waste. In connection with the guidelines of the Council Directive 1999/31/EC of 26 April 1999, it is required to enforce environmental regulations and regulations concerning the monitoring of pollution in the area of landfills, considering the aquatic environment, including seepage waters. Such an obligation in EU countries includes monitoring at landfills during both the operational and post-operational phase (Magrinho et al. 2006).

Leachate may exert a significant influence on the condition of the environment, which is formed under the influence of the infiltration of rainwater in the deposit of stored waste which is associated with the percolation through the waste, organic, inorganic, colloidal, pathogenic, and other contaminated materials, which are also transferred (Zin et al. 2012). Leachate is a potential source of contamination of surface water, groundwater, and soil (Barbieri et al. 2014; Naveen et al. 2018; Shershneva et al. 2017). Therefore, areas close to landfills have a greater chance of water pollution due to the possible contamination source of leachate originating from the dump sites (Samadder et al. 2017). The effect of landfill leachate on surface water and groundwater has been reported by some researchers (Abu 2010; Guan et al. 2014; Quaghebeur et al. 2013).

Leachate is the longest emitted pollution from landfills and is considered dangerous when heavily polluted and remaining without capture and treatment (Amuda 2005). Their chemical composition changes continuously over time and depends mainly on the type of waste deposited and the method of exploitation of the deposit itself (Peng et al. 2008; Kulikowska 2009). The quality of the leachate is significantly influenced by the age of the deposited waste (Lee et al. 2010). In many countries, including Poland, water quality monitoring is based on physical and chemical analyses (Drobniewska et al. 2007). Assessing the influence of the existing landfills on surface water and groundwater quality is not an easy task, as the selection of indicators should be identical for the two types of water as well as for leachate.

Yusof et al. (2009) recognised the influence of leachate on surface water on the basis of chemical composition. Leachate from municipal solid waste landfill sites is a highly concentrated complex liquid waste containing dissolved organic and inorganic compounds, such as ammonia, calcium, magnesium, sodium, chlorides, and heavy metals, such as chromium, copper, lead, iron, and nickel (Longe and Balogun 2010). Their concentration in leachate and water depends on the composition of the deposited waste (Alker et al. 1995).

However, particular importance is attached to groundwater quality within landfills, as the presence of organic matter and ammonia, which are typical water pollutants due to their strong mobility, can have significant effects on this environment (Mao et al. 2018). In general, the degree of risk of water pollution depends on the structure of the landfill, the type and number of deposits, and the water geological and hydrographic conditions of the location of the landfill (Rapti-Caputo et al. 2006).

Studies of the influence of deposited waste on the aquatic environment may be conducted using physicochemical indicators, considering the ambient temperature and the temperature of the leachate and surface water and groundwater as well as their level and surface flow rate. The objective of the study was to assess the influence of an old municipal waste storage site located near a watercourse on the aquatic environment in general, using a wide range of indicators.

## Materials and methods

### Landfill area

**Fig. 1:**
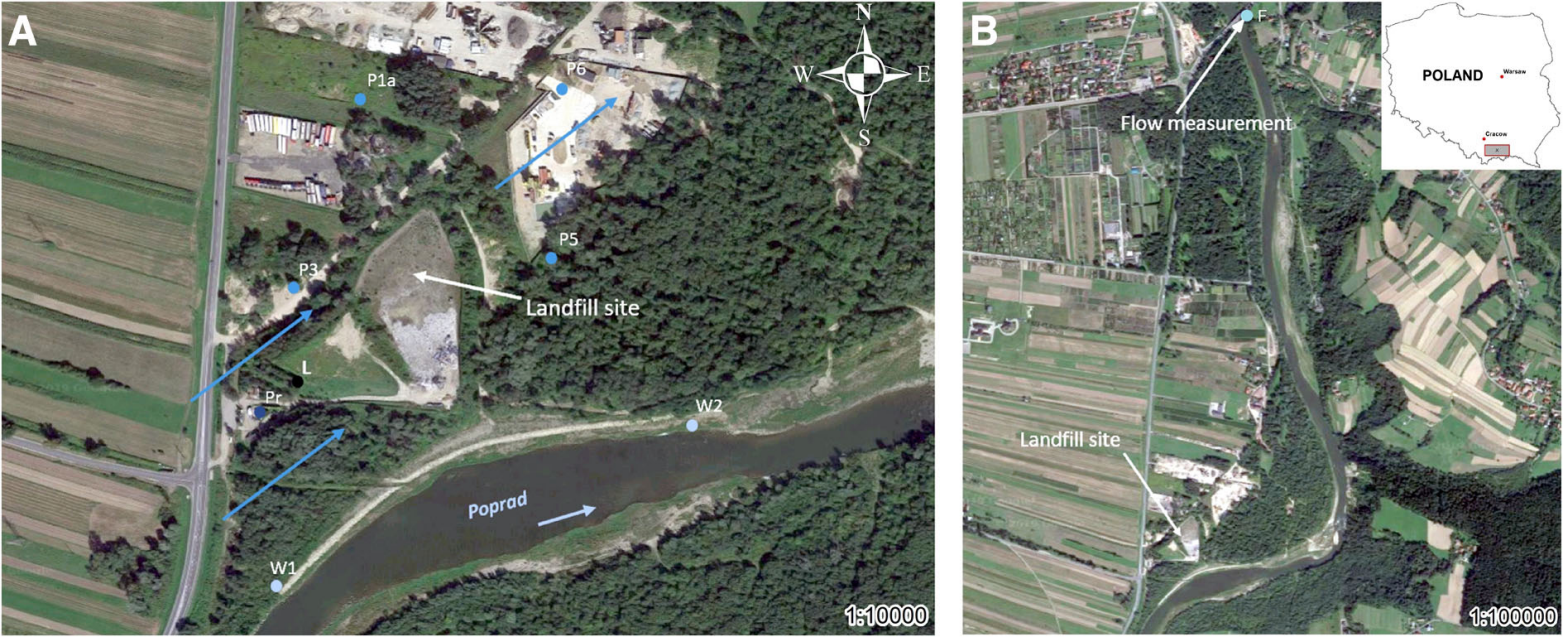
Location of examined points in the surroundings of the municipal solid waste landfill site in Stary Sącz (Southern Małopolska, Poland): **a** points for surface water intake (W1, W2), groundwater intake (P1a, P3, P5, and P6), leachate intake (L) (including air measurement), and atmospheric precipitation measurement (Pr); direction of groundwater flow is shown with blue arrows and direction of surface water flow is shown with bright blue arrows ; **b** point measuring the flow of the Poprad River (F)

The landfill in S town is located in the southern part of the Lesser Poland region (Fig. 1). Its operation began in 1999, and the shutdown will take place after the landfill quarter has been filled to the planned level. The examined landfill covers an area of almost 1.50 ha and is located on the first left-bank floodplain and over the floodplain terrace of the Poprad River, about 100 m from this river. Poprad River originates in the Slovak part of the High Tatras, flows into the Dunajec River below the landfill, and is classified as a mountain river (Hawryluk and Cholewa 2016). Ford et al. (2011) demonstrated that old landfills were located in floodplains near watercourses.

The landfill site is located within the Magura Nappe, which is built by chalk and Palaeogene aged settlements (i.e. mutually stratifying sands and slates and typical flysch formations). There are tertiary formations of the deeper substrate covered with Holocene river formations in the form of pebbles, gravel, sand-gravel mixes, and sand with thin mud pads.

In this area, groundwater is found in flysch and quaternary formations. In flysch formations, water is contained in the sandstone layers of the bedrock. The amount depends on the size of the inter-joining sandstone cracks and the porosity of the sandstone. In quaternary formations, the main aquifer is found in Holocene stone and gravel formations of the Poprad River terrace. These waters have a hydraulic connection with the waters of the river, the valley of which is a system that drains underground waters flowing down the mountain slopes. Generally, groundwater runoff takes place in the direction of the Poprad River from southwest to northeast (Fig. 1).

At the analysed landfill, residue is deposited from the recovery of waste material from several municipalities located in the vicinity of the landfill. This type of waste management generally reduces the amount of landfilled waste. Waste deposited at municipal solid waste landfill sites is spread and thickened by a bulldozer in thin layers of 0.3–0.5-m thick to a height of 1.2 m, followed by a systematic transfer of an insulating layer with a maximum thickness of 0.3 m.

The landfill site consists of two sectors: sector I (reclaimed with a capacity of 86.268 m^3^) and sector II (operational phase with an operating capacity of 134.932 m^3^). The area adjacent to the landfill is slightly sloping in a northeastern direction. Within it, there is a rampart 2.0–5.0-m high. The bottom of sector II of the landfill was sealed with synthetic insulation in the form of Bentomat, 2.5-mm thick HDPE geomembranes, and geotextile to protect the ground and water environment. The leachate at the landfill is captured with filters with a diameter of 100 mm and collective drainage with a diameter of 200 mm. They are stored in a tank with a capacity of 18.3 m^3^.

### Examined points

Surface water samples were taken within 12 months in the Poprad riverbed at two points: W1 above the landfill and W2 below the landfill. There are four piezometers within the landfill from which groundwater samples were taken for qualitative and quantitative tests. Piezometer P3, located on the inflow of groundwater to the northwest of the landfill, was considered a reference point. Another three piezometers below the landfill (P1a, P5, and P6) are located at the outflow of groundwater to the west, northeast, and east of the landfill, respectively. The leachate water produced at the landfill was collected for tests in a reservoir located in the southwestern part of the landfill (Fig. 1).

### Scope of research

The study uses the results from our own tests and from monitoring tests made available by the landfill administrator. In our own studies (August 2017 to July 2018), the temperature (including ambient temperature), the pH value, conductivity, concentration of dissolved oxygen (DO), and ammoniacal nitrogen (NH_4_-N) were determined in surface water, groundwater, and leachate from landfill.

The results obtained from the user of the landfill included the determination of biochemical oxygen demand (BOD_5_), chemical oxygen demand (COD), total organic carbon (TOC), total nitrogen (N), nitrate nitrogen (NO_3_-N), polycyclic aromatic hydrocarbons (PAHs), copper (Cu), cadmium (Cd), chromium (Cr^+6^), and mercury (Hg) (Kayode et al. 2018). The survey covered four quarters from August 2017 to July 2018. These heavy metals are often used for leachate toxicity testing (Przydatek 2019). In addition, measurements of the surface water level (Poprad River) were conducted independently using a GPS Spectra Precision SP60 GNS geodetic receiver and a Leica NA320 optical levelling device with rectification and measuring staff, and groundwater was measured in piezometers using a hydrogeological marrow. For all research points, the data (in metres above sea level) were determined before the investigation. The reaction, specific electrolytic conductivity, DO content, and temperature of the surface water, groundwater, and leachate water samples taken in the landfill area were determined immediately after collection. For this purpose, a portable multifunctional meter with glass electrodes was used after calibration.

The quality of groundwater in the tested piezometers was determined in accordance with the Regulation of the Minister of the Environment (21 December 2015) on the method of criteria and of assessment of the condition of bodies of groundwater (21 July 2016) and on the method of classification of the condition of bodies of surface water and environmental quality standards for priority substances (Regulation of the Minister of the Environment 2016). The results of the leachate water tests from the landfill were compared with the values included in the Regulation of the Minister of the Environment on 18 November 2014 on the conditions to be met when discharging sewage into water or soil. The collected results of physicochemical indicators of surface water, groundwater, and leachate status and the meteorological elements were used for statistical analysis and for drawing conclusions aimed at assessing the influence of the landfill on the water quality in its surroundings.

### Water sampling

Surface water samples were taken with a telescopic bucket. Then, after previous test pumping, groundwater samples were taken from a given piezometer using a portable pump. Raw leachate from the landfill was sampled using a bucket from a collecting well located on the landfill site (Fig. 1). The samples were placed in sterilised polyethylene containers. As part of the research, each month from August 2017 to July 2018, the samples of surface water, groundwater, and leachate water were collected for the measurement of temperature, pH, conductivity, and the content of DO and ammoniacal nitrogen (NH_4_-N). In each quarter between July 2017 and August 2018, on behalf of the landfill administrator, samples were taken four times for determining BOD_5_, COD, TOC, PAHs, total N, NO_3_-N, Cu, Cd, Cr^+6^, and Hg. The minimum sample amount was 500 mg L^−1^. Samples for analysis were delivered on the same day or within a maximum of 24 h to an accredited testing laboratory (chemical indicators), where the analysis was conducted according to accredited methods with quality control and confidence (APHA 2007). The quality of the analytical measurements was investigated using quality assurance (QA)/quality control (QC) samples (i.e. method blank and control samples; Cathum and Sabik 2001). In addition, a detailed study was conducted on field controls.

### Physicochemical analysis

Directly after sampling the surface water, groundwater, and landfill leachate, under field conditions, the temperature (including ambient temperature), reaction, specific electrolytic conductivity, and DO content in the electro-chemical method were determined. These indicators were used by Thomsen et al. (2012). A portable multifunction meter with glass electrodes (CX-461), which was calibrated each time before the tests, was used for the determination. Each result was based on the average of the three measurements. The samples were transported to the laboratory under refrigerated conditions (at 4 °C), without light access, with minimal exposure to oxygen (Ward et al. 2005).

In the accredited chemical laboratory, the following content was determined: BOD_5_, COD, N, NO_3_-N, NH_4_-N, Cu, Cd, Cr^+6^, Hg, PAHs, and TOC. Heavy metals like Cu, Cd, Cr^+6^, and Hg were determined by atomic absorption spectroscopy (AAS) and NH_4_-N was determined using the spectrophotometric method. Nitrates were determined using the colorimetric method. Biochemical oxygen demand (BOD_5_) was determined by dilution and grafting with allylthiourea, and COD was determined using the dichromatic method.

Water and leachate samples for analysis were initially mineralised with nitric acid and hydrogen peroxide at elevated temperatures. Mineralisation was conducted in open vessels using a heating plate. After mineralisation, the samples were transferred quantitatively and seeped through a tissue filter. The mineralisation vessel was washed with water, and the solution was decanted after washing and attached to the same vessel. The extract prepared in this way was ready to be determined using atomic emission spectrometry with inductively excited plasma (ICP-OES). Mercury in the tested samples was determined using the AAS technique, in which the phenomenon of the absorption of electromagnetic radiation by elements in the atomic form was used. The determination was based on the introduction of Hg vapours into a special absorption cell through which ultraviolet radiation with a wavelength of 253.7 nm was transmitted. The radiation generated by the lamp was divided into two streams: one part went into a chamber with pairs of Hg, and the other stream served as a reference based on the difference in intensity between the streams, and the Hg content was calculated. The AAS technique for determining heavy metals in leachate was also used by Olivero-Verbel et al. (2008). Laboratory analyses were performed twice (repeated) in the cases of exceeding the limit values, obtaining results outside the calibration curve, or obtaining unusual results for a given matrix.

Biochemical oxygen demand (BOD_5_) was based on determining the O_2_ consumption during the 5-day incubation in the process of organic compound mineralisation. Moreover, BOD_5_ is the result of the difference in DO content at the beginning of the test and after 5 days. The COD was determined using K_2_Cr_2_O_7_ and an oxidisability of 100 mg L^−1^. The oxidation process was conducted under strict conditions and time. After oxidation, the amount of the remaining oxidant was determined.

The amount of ammonium nitrogen was determined using the spectrophotometric method. The method is based on the reaction of ammonium ions present in the sample added to a gas segmented carrier stream with alkaline hypochlorite (ClO^-^) previously released from sodium dichloroisocyanurate. The resulting chloramine reacts as a catalyst with salicylate in the presence of nitroprusside at temperatures of 37 to 50 °C to form a blue-green indophenol dye, which is then quantified in a flow photometer at wavelengths of 640 to 660 nm. The sample was analysed directly on a flow analyser with spectrophotometric detection (CFA). Nitrite-nitrogen was determined using the colorimetric method. The concentration of nitrate nitrogen was calculated as the total concentration of nitrate and nitrite-nitrogen and the primary concentration of nitrate nitrogen. The total nitrogen is the sum of organic, ammonium, nitrate, and nitrite-nitrogen. The content of the leachate chemical contamination index was determined with the accuracy of milligrams per litre.

### Precipitation, leachate amounts

The data on the monthly sums of precipitation in the period from August 2017 to July 2018 came from the investigated landfill. The monthly precipitation in the landfill site was determined using the Hellmann 1500 Lambrecht rain gauge (Fig. 1).

The amount of leachate generated at the landfill in the analysed period was determined on the basis of the amount of leachate delivered to the sewage treatment plant by road transport in individual months between August 2017 and July 2018.

### Waste amounts

The landfill administrator provided data on the quantities of deposited waste for the period from August 2017 to July 2018, the weight of which was determined using electronic scales with a capacity of 30 Mg. In the research period, operational data on flow rates recorded on the day of the research were obtained from a water gauge from the Institute of Meteorology and Water Management (IMWM) located on the Poprad River (N 49° 34´ 07˝ E 20° 39´ 35˝), approximately 2 km north of the examined facility in the S direction (Fig. 1). Moreover, in the landfill area on the day of sampling, the atmospheric air temperature was measured using a multifunctional meter (CX-461) at each of the research points (W1, W2, P1a, P3, P5, and P6), on the basis of which the average value for a given day was determined.

### Statistical analysis

For the results of the studied water quality, including physical and chemical elements, the following statistical parameters were determined: minimum value, maximum value, and arithmetic mean. For the calculation of certain values of statistical parameters (BOD_5_, COD, TOC, total N, NO_3_-N, PAHs, Cu, Cd, Cu, Cr^+6^, and Hg), a measurement result at half of the quantification limit was used when the value of the water indicators in the sample was below the quantification limit (i.e. the output signal or concentration value above which it can be stated with some certainty that the sample is different from a blank sample).

The results of surface water and groundwater levels, water and leachate sample temperatures, atmospheric air, reaction determination, EC, DO, NH_4_-N content and precipitation, and amount of leachate and waste were subjected to statistical analysis considering the following characteristic values: minimum, maximum, mean, and standard deviation. The Shapiro-Wilk test was used to check the normality of the distribution of the analysed data. Pearson’s coefficient of linear correlation (*n* = 12) was used to check the relationships between the listed variables. The test probability of *p* < 0.05 was assumed to be significant. To estimate the significance of differences in concentrations of the examined indicators of surface water and groundwater above and below the municipal waste storage site, Levene’s parametric test of multiple comparisons of average ranks, which requires a normal distribution, was applied. Extreme values, mean values, and the standard deviation are presented on box plots for selected elements that differ significantly from one point to another. Parametric tests were applied due to the normal distribution of most of the analysed indices, including the physicochemical ones. Statistica 12 (StatSoft Poland, StatSoft, Inc., USA) software was used for statistical analysis.

Several methods have been used to discriminate between natural and anthropogenic effects, primarily based on geo-chemical principles (Griffioen et al. 2008; Hinsby et al. 2008) and data-driven statistics (Molinari et al. 2012; Wendland et al. 2005).

## Analysis of results

### Quantitative analysis of deposited waste, leachate, and selected meteorological elements

Between August 2017 and July 2018, the amount of municipal waste deposited at the S landfill ranged from 25.81 to 265.04 Mg with an average of 116.29 Mg. The highest amount of waste was in March 2018. Moreover, 1.396 Mg of waste was deposited within 12 months (between August 2017 and July 2018) of the landfill operation. The total amount of identifiable waste in the years 2009 to 2018 amounted to 8.033 Mg, with a daily accumulation below 10 Mg.

The amount of leachate collected at the landfill ranged from 0 to 634 m^3^ with an average of 329.08 m^3^. The highest amount of leachate in the landfill occurred in June and the highest precipitation of 173.5 mm occurred in September 2017 with an overall average of 66.18 mm. The size of these parameters over the course of 12 months was characterised by a downward trend.

**Fig. 2:**
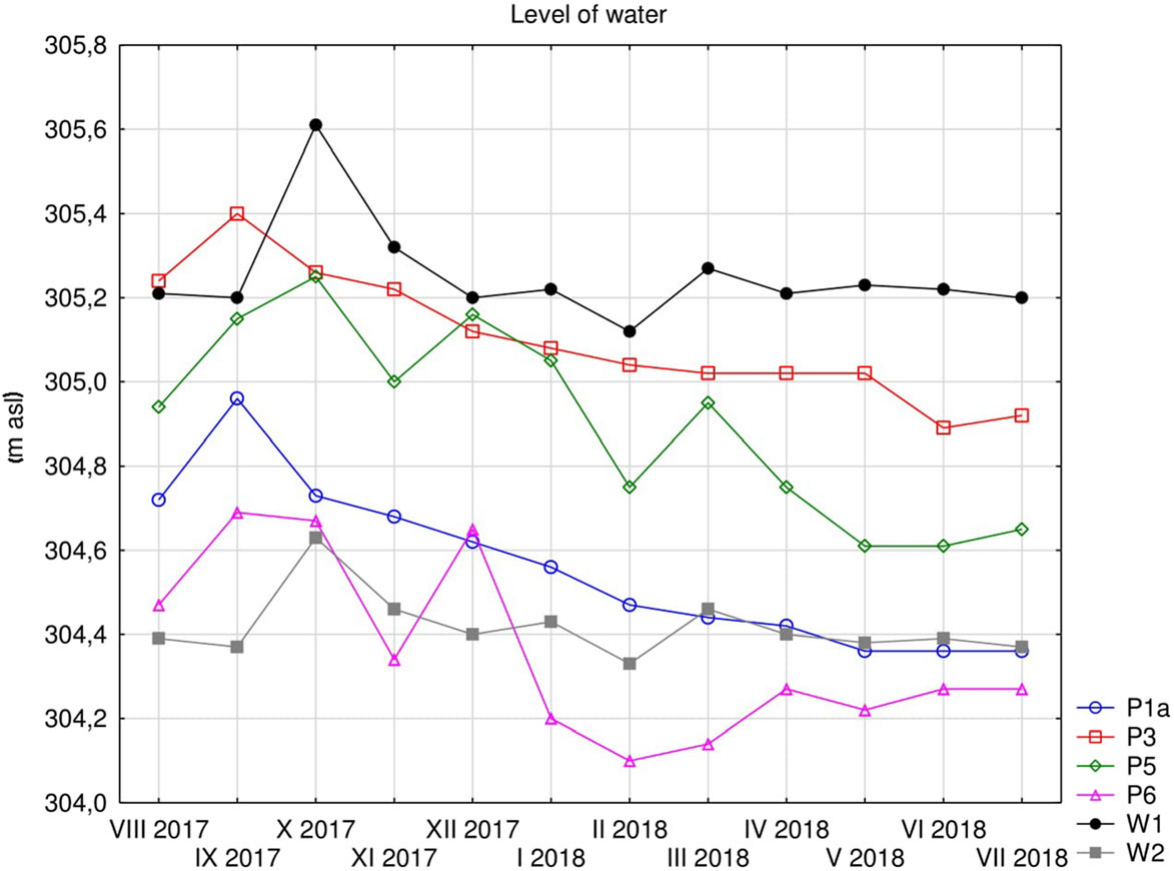
Level of water

Another examined indicator related to the flow of water in the Poprad River was characterised by the variability in the range of 9.9 to 55 m^3^ h^−1^, which was at the highest in October. The level of surface and groundwater was characterised by variability. The highest mean level was observed on the inflow of groundwater above the landfill (mean altitude of 305.10 m asl). The highest water inflow was at P3 (altitude 305.40 m asl) in September, and the highest (altitude 305.25 m asl) was in October at P5. At this point, the groundwater level was at one of the shallowest levels at 2.5 m in July with an average depth of 3.26 m below ground level. Groundwater in the piezometric borehole P1a (mean depth of 4.10 m below ground level) was the deepest. The lowest level was found in the period of three months from May to July (Fig. 2). Another index of atmospheric air temperature during the research period showed fluctuations between − 1.7 °C and 25.6 °C in February and May 2018, respectively Table 1.

**Table 1:** Scope and average values of physicochemical elements of groundwater quality class

Psyhico-chemical elements		Study points												Classification quality of groundwater in accordance with Regulation ME (2015)				
	Unit	P1a		P3		P5		P6		P1a	P3	P5	P6					
		Min	Max	Min	Max	Min	Max	Min	Max	Average	Average	Average	Average	I	II	III	IV	V
Reaction	(pH)	6.6	7.4	6.4	7.1	6.2	7.4	5.5	7.4	7.0	6.9	6.7	6.8	6.5–9.5			< 6.5 or > 9.5	
Temperature	(°C)	5.6	14.4	7.1	14.7	6.3	14.5	6.3	16.1	10.44	11.35	10.59	10.99	< 10	12	16	25	
EC	(μS cm^−1^)	186	906	167	1003	235	1099	134	1158	777	745	840	666	700	2500	2500	3000	> 3000
Dissolved oxygen	(mg L^−1^)	1.02	6.44	0.00	6.57	0.00	5.18	0.96	4.89	3.83	2.50	1.90	2.45	> 1	0.5–1	< 0.5	< 0.5	< 0.5
Ammoniacal nitrogen	(mg L^−1^)	0.00	0.60	0.03	10.07	0.02	0.12	0.03	1.13	0.095	1.34	0.05	0.22	0.5	1	1.5	3	> 3

### Quantitative analysis of surface, underground, and leachate waters

The analysis of water quality indicators of the Poprad River flowing in the vicinity of the surveyed municipal solid waste landfill sites on the basis of the Regulation of the Minister of the Environment (RME) (2016) showed that most of the examined physicochemical elements meet the standards of very good quality water at class I, except for COD (Table 2). Based on the mean values of this designation at points W1 and W2 (mean concentration was 14.5 and 15.75 mg L^−1^, respectively), surface water based on the RME (2016) was classified to class II due to exceeding the limit value of class I. Otherwise, the selected physicochemical elements of surface water (temperature, pH, EC, DO, and NH_4_-N) did not show any deterioration of the quality class for the water (Table 3).

**Table 2:** Scope and average values of physicochemical elements of surface water quality class—monitoring tests

Physicochemical element	Unit		Study points					Classification quality of surface water with accordance with Regulation ME (2016)	
		W1		W2		W1	W2		
		Min	Max	Min	Max	Average		I	II
Lead	(mg L^−1^)	< 0.001	< 0.005	< 0.001	0.001	0.002	0.002	0.0072	
Cadmium	(mg L^−1^)	< 0.00030	< 0.03	< 0.00030	0.0005	0.004	0	0.00045–0.015	
Copper	(mg L^−1^)	< 0.0020	0.024	< 0.0020	0.024	0.007	0.009	0.05	
Zinc	(mg L^−1^)	< 0.050	0.078	< 0.03	0.112	0.033	0.044	1	
Chromium(VI)	(mg L^−1^)	< 0.010	< 0.030	< 0.010	< 0.010	0.008	0.005	0.05	
Mercury	(mg L^−1^)	< 0.000050	0.05	< 0.000050	0.05	0.019	0.019		
TOC	(mg L^−1^)	< 1.0	3.8	< 1.0	5.7	3	2.2		
COD	(mg L^−1^)	23	23	10	23	14.5	15.75	≤ 10	≤ 20
BOD_5_	(mg L^−1^)	1.8	2.2	1.8	2.3	1.75	1.78	≤ 3	6
Nitrate nitrogen	(mg L^−1^)	1.36	5.5	1.49	5.2	3.47	3.48		
Total nitrogen	(mg L^−1^)	0.42	2.6	0.49	2.5	1.44	1.42	5	10
PAHs	(μg L^-1^)	< 0.030	< 0.036	< 0.030	< 0.036	0.017	0.017

**Table 3:** Scope and average values of physicochemical elements of surface water quality class

Physicochemical element		Study points						Classification quality of surface water in accordance with Regulation ME (2016)				
		W1		W2		W1	W2					
	Unit	Min	Max	Min	Max	Average	Average	I	II	III	IV	V
Flow	(m^3^ s^−1^)	10	55	10	54	22.33	22.29					
Reaction	(pH)	7.7	8.5	7.7	8.5	8.2	8.2	6.0–8.5	6.0–9.0			
Temperature	(°C)	− 0.1	22.1	− 0.1	20.1	10.11	10.06	≤ 22	24			
EC	(μS cm^−1^)	246	705	247	789	358.1	397.5	≤ 1000	1500			
Dissolved oxygen	(mg L^−1^)	5.88	11.91	6.22	10.95	8.83	8.85	≥ 7	5			
Ammoniacal nitrogen	(mg L^−1^)	0.02	1.41	0.02	0.60	0.20	0.13	0.78	1.56			

The analysis of groundwater quality in piezometers located in the landfill area showed that most of the examined physicochemical elements meet the standards of very good water quality at class I. Below the landfill, due to its nitrate content in piezometer P5 (mean concentration of 14.75 mg L^−1^), groundwater was classified as good, at class II. A similar result of the mean nitrate content was achieved in the piezometer at the inflow of water in piezometer P3. However, the quality of groundwater below the storage site deteriorated in P1a to a satisfactory class III groundwater quality due to the average concentration of NO_3_-N, which was 28 mg L^−1^ as a result of natural processes taking place in groundwater or anthropogenic factors (Table 4). Generally, out-of-class groundwater quality at the outflow in three piezometers was caused by exceeding the limit value by average concentrations of the determined PAHs, classifying water as poor quality (class V). Only in piezometer P3, the average concentration did not exceed 0.0005 mg L^−1^ and showed that the tested waters from this well were classified at an unsatisfactory quality (class IV) of groundwater. The mean values of groundwater temperature at the four tested piezometric points ranged from 10.44 to 11.35 °C. Such results showed that the acceptable level of purity for class I was exceeded at the highest mean value in P3. Similarly, the average EC values in the range of 745 to 840 μS cm^−1^ in the tested piezometers confirmed that the first class of purity was exceeded. The highest average value of this indicator occurred at the point below the P5 storage site. Alternatively, the highest average concentration of 1.34 mg L^−1^, determined as NH_4_-N, occurred on the inflow of these types of water to the landfill, which resulted in the classification of the water into class II (Table 5). At the outflow, the mean concentrations of NH_4_-N were within the range of 0.05 to 0.22 mg L^−1^.

**Table 4:** Scope and average values of physicochemical elements of groundwater quality class—monitoring tests

Physicochemical elements		Study points												Classification quality of groundwater in accordance with Regulation ME (2015)				
	Unit	P1a		P3		P5		P6		P1a	P3	P5	P6					
		Min	Max	Min	Max	Min	Max	Min	Max	Average				I	II	III	IV	V
Lead	(mg L^−^1)	< 0.001	< 0.001	< 0.001	< 0.010	< 0.001	< 0.01	< 0.001	< 0.01	0.001	0.002	0.003	0.003	0.01	0.025	0.1	0.1	> 0.1
Cadmium	(mg L^−^1)	< 0.00030	< 0.0004	< 0.00030	< 0.0004	< 0.00030	< 0.0004	< 0.00030	< 0.0004	0.0002	0.0002	0.0002	0.0002	0.001	0.003	0.005	0.01	> 0.01
Copper	(mg L^−^1)	0.0037	0.0037	0.002	0.0037	0.021	0.021	0.023	0.023	0.002	0.012	0.007	0..08	0.01	0.05	0.2	0.5	> 0.5
Zinc	(mg L^−^1)	0.066	0.066	0.034	0.066	0.037	0.037	0.037	0.037	0.039	0.028	0.026	0.029	0.05	0.5	1	2	> 2
Chromium(VI)	(mg L^−^1)	< 0.01	< 0.01	< 0.01	< 0.01	< 0.01	< 0.01	0.005	0.005	0.005	0.005	0.005	0.005	0.01	0.05	0.05	0.1	> 0.1
Mercury	(mg L^−^1)	0.00005	0.00005	0.00005	0.00005	0.00005	0.00005	0.00005	0.00005	0.00003	0.00003	0.00003	0.00003	0.001	0.001	0.001	0.005	> 0.005
TOC	(mg L^−^1)	1.1	1.1	1.7	2.4	1.9	2.4	1.6	1.7	0.90	1.53	1.20	0.95					
COD	(mg L^−^1)	< 3.0	< 10	50	50	< 10	39	< 10	10	3.83	23.33	16	11.25					
BOD_5_	(mg L^−^1)	< 0.5	< 3.0	< 0.5	5.2	< 0.5	14	< 0.5	0.5	0.67	2.32	4	0.94					
Nitrate nitrogen	(mg L^−^1)	24.9	30.3	3.73	28.8	10.4	17.8	1.7	2.4	28	15.01	14.75	2.15	10	25	50	100	> 100
Total nitrogen	(mg L^−^1)	5.91	7.12	3.65	6.53	2.5	8.04	0.542	1.68	6.52	4.42	4.43	0.79					
PAHs	(μg L^−1^)	< 0.000036	< 0.030	< 0.000036	0.000472	< 0.000036	< 0.03	< 0.000036	< 0.03	0.008	0.005	0.008	0.008	0.0001	0.0002	0.0003	0.0005	> 0.0005

**Table 5:** Scope and average values of physicochemical elements of groundwater quality class

Physicochemical elements		Study points												Classification quality of groundwater in accordance with Regulation ME (2015)				
	Unit	P1a		P3		P5		P6		P1a	P3	P5	P6					
		Min	Max	Min	Max	Min	Max	Min	Max	Average				I	II	III	IV	V
Reaction	(pH)	6.6	7.4	6.4	7.1	6.2	7.4	5.5	7.4	7.0	6.9	6.7	6.8	6.5–9.5			< 6.5 or > 9.5	
Temperature	(°C)	5.6	14.4	7.1	14.7	6.3	14.5	6.3	16.1	10.44	11.35	10.59	10.99	< 10	12	16	25	
EC	(μS cm^−1^)	186	906	167	1003	235	1099	134	1158	777	745	840	666	700	2500	2500	3000	> 3000
Dissolved oxygen	(mg L^−1^)	1.02	6.44	0.00	6.57	0.00	5.18	0.96	4.89	3.83	2.50	1.90	2.45	> 1	0.5–1	< 0.5	< 0.5	< 0.5
Ammoniacal nitrogen	(mg L^−1^)	0.00	0.60	0.03	10.07	0.02	0.12	0.03	1.13	0.095	1.34	0.05	0.22	0.5	1	1.5	3	> 3

The results of the leachate testing of the analysed landfill site with respect to physiochemical indicators did not show that the limit values were exceeded (Regulation 2014). The leachate waters reaction was close to neutral at a pH of 7.4, and the mean leachate temperature did not exceed 10 °C. The PEW remained within the range of 869 to 4371 μS cm^−1^, while the dissolved oxygen content oscillated within the range of 11.08–200.8 mg L^−1^. The average concentration of total nitrogen was 12.08 mg L^−1^, and the nitrate nitrogen was lower than the previous concentration by more than 8 mg L^−1^. On the other hand, the content of ammonium nitrogen ranged from 0.3 to 9.43 mg L^−1^ with an average content of 2.09 mg L^−1^, which was significantly lower than the listed biogens. The highest mean contamination concentration of 59.25 mg L^−1^ in the leachate was determined using COD. The mean content of TOC 14.57 mg L^−1^ was lower, as were PAHs with an average content of 0.008 mg L^−1^. Among the examined heavy metals, the highest concentration showed zinc at 0.12 mg L^−1^, whereas the other results of heavy metals did not exceed the concentration of 0.030 mg L^−1^.

### Statistical comparative analysis of test results

**Fig. 3:**
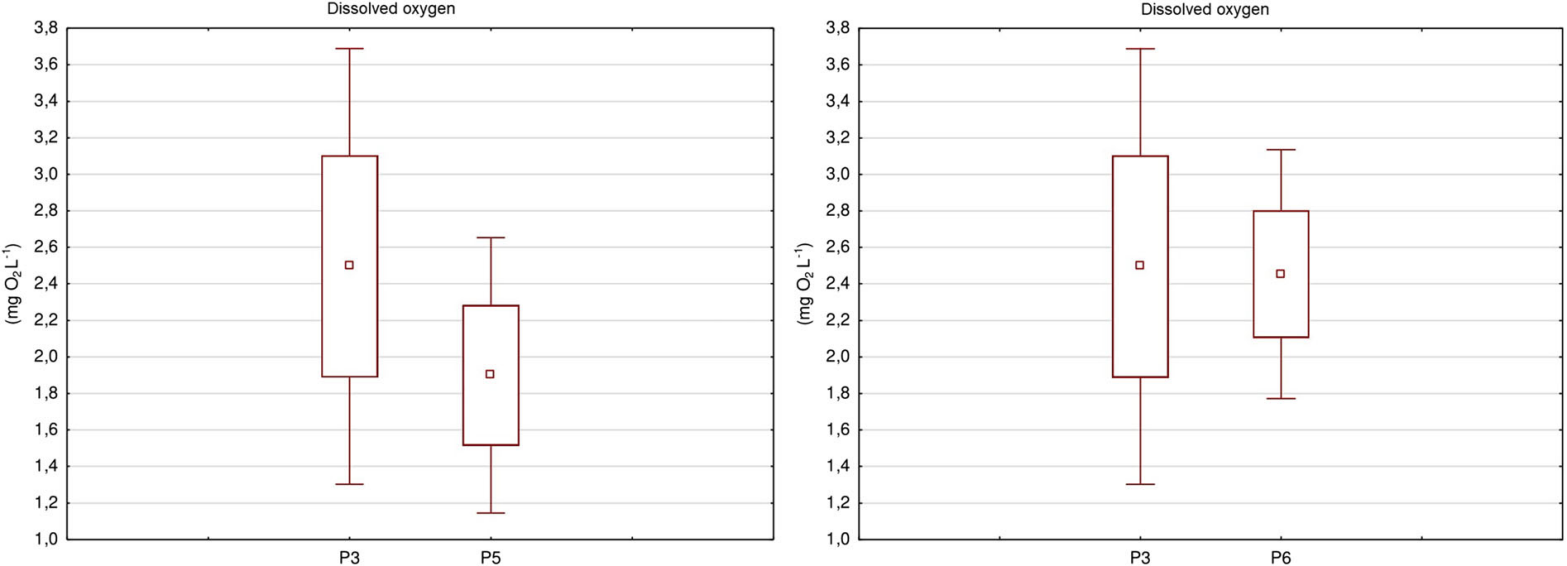
Difference between points of groundwater

Statistical comparative analysis of physicochemical elements in groundwater conducted with the Levene’s parametric test showed that, out of the five examined indicators, two (i.e. DO and NH_4_-N) differ significantly statistically between piezometers (Table 6). These differences concern the piezometer located above the landfill and the piezometers below the landfill. The values of DO were statistically significantly higher in piezometer P3 than in piezometers P5 (*p* = 0.03) and P6 (*p* = 0.02) (Fig. 3). In piezometer P3, the concentration of NH_4_-N was higher than in piezometer P5.

**Table 6:** Comparison of physicochemical index between examined points using Levene’s test

			Results of Levene’s test	
Physicochemical index	Examined points	Average	Test value	Probability test (*p*)
pH	River			
	W1	8.2	0.005	0.94
	W2	8.2		
	Piezometer			
	P3	6.85	0.65	0.43
	P5	6.72		
	P3	6.85	3.31	0.08
	P6	6.83		
Temperature (°C)	W1	10.11	0.05	0.83
	W2	10.06		
	P3	11.35	0.10	0.75
	P5	10.59		
	P3	11.35	0.40	0.54
	P6	10.99		
EC (μS cm)	W1	358	0.92	0.35
	W2	397		
	P3	745	0.17	0.69
	P5	840		
	P3	745	0.16	0.69
	P6	666		
Dissolved oxygen (mg L^−1^)	W1	8.83	0.06	0.80
	W2	8.85		
	*P3*	2.50	5.18	*0.03*
	*P5*	1.90		
	*P3*	2.50	5.89	*0.02*
	*P6*	2.45		
Ammoniacal nitrogen (mg L^−1^)	W1	0.20	1.32	0.26
	W2	0.13		
	*P3*	1.34	4.54	*0.04*
	*P5*	0.05		
	P3	1.34	3.34	0.08
	P6	0.22		

Statistical values in italics statistically significant differences at *p* < 0.05

**Fig. 4:**
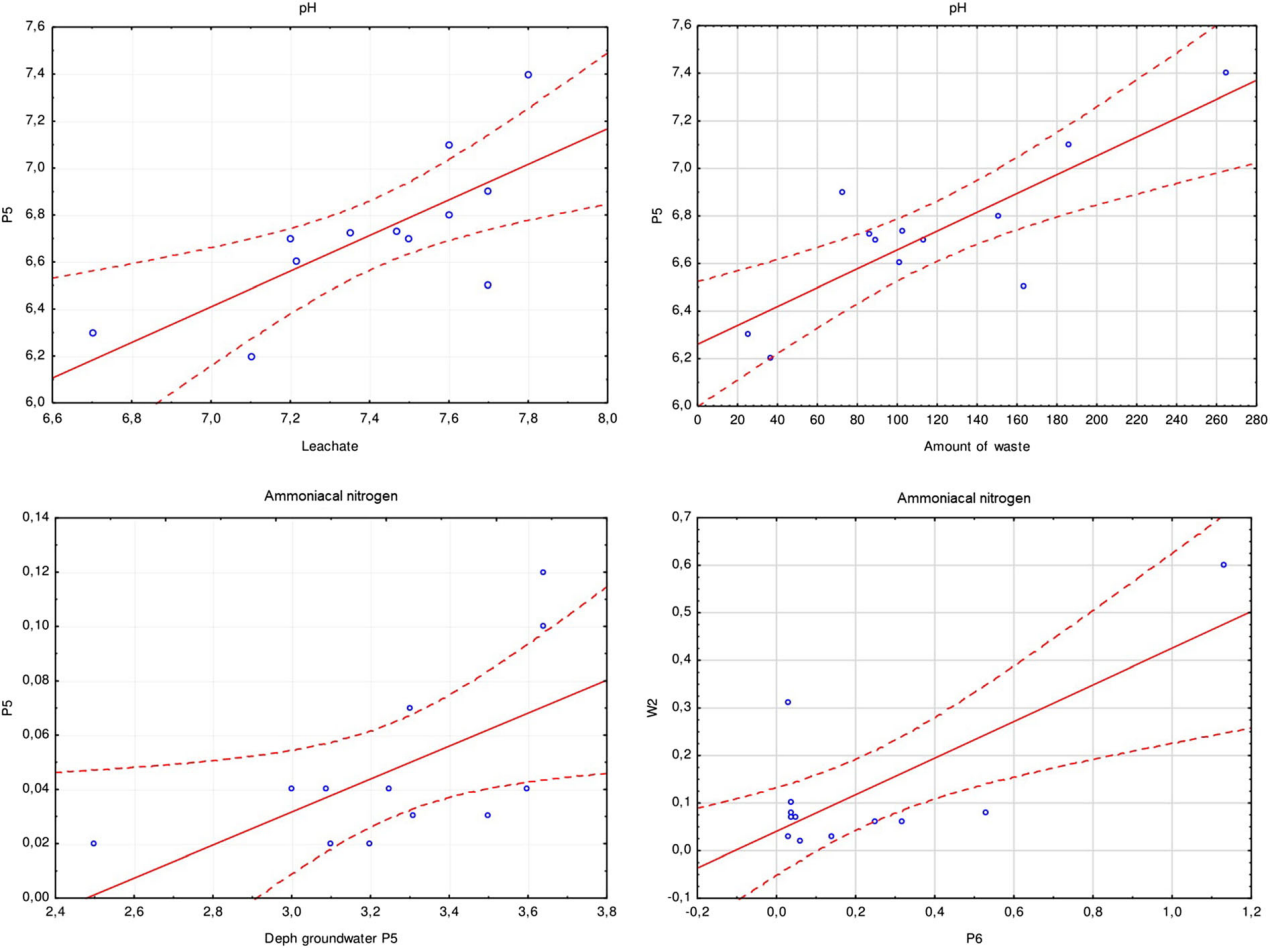
Correlation between selected points

To assess the influence of the landfill on the physicochemical state of the surface and groundwater, a correlation (positive) relationship between water in the Poprad River and piezometers below the landfill and four variables, including leachate, air temperature, precipitation, and waste, was analysed. A very high correlation was observed between water in piezometer P5 and the leachate in terms of pH (*r* = 0.736, *p* < 0.05) (Fig. 4) and the quantity of waste deposited (*r* = 0.768, *p* < 0.05) (Table 7). The concentration of NH4-N in groundwater in P5 was statistically significantly correlated with the depth of groundwater deposits (*r* = 0.609, *p* < 0.05). However, surface water at point W2 showed a statistically significant relation with water in piezometer P6 below the landfill, considering ammonium nitrogen (*r* = 0.749, *p* < 0.05**)** (Fig. 4). The highest concentrations of NH_4_-N at the analysed points occurred in August. The above analysis, based on the Pearson correlation, shows that the strongest relationship occurred between water in the piezometer below the landfill and waste when comparing the pH value and amount of waste.

**Table 7:** Correlation between selected indicators of examined points’ location around of the landfill site

Variable	Unit	W2	P5	P6	Leachate	Amount leachate	Air temperature	Precipitation	Waste
W2	(pH)		0.317	0.370	0.484	−0.114	0.135	− 0.048	0.180
P5	(pH)	0.317		0.343	*0.736*	− 0.525	− 0.391	− 0.446	*0.815*
P6	(pH)	0.370	0.343		0.037	0.324	0.488	− 0.020	− 0.101
Leachate	(pH)	0.484	*0.736*	0.037		*− 0.725*	− 0.477	*− 0.735*	*0.768*
Amount leachate	(m^3^)	− 0.114	− 0.525	0.324	*− 0.725*		*0.723*	0.536	*− 0.667*
Air temperature	(°C)	0.135	− 0.391	0.488	− 0.477	*0.723*		0.390	− 0.468
Precipitation	(mm)	− 0.048	− 0.446	− 0.020	*− 0.735*	0.536	0.390		− 0.571
Waste	(Mg)	0.180	*0.815*	− 0.101	*0.768*	*− 0.667*	− 0.468	− 0.571	
W2	(°C)		0.566	*0.604*	*0.840*	*0.876*	*0.941*	0.507	− 0.545
P5	(°C)	0.566		*0.856*	*0.744*	*0.714*	0.407	*0.770*	*− 0.764*
P6	(°C)	*0.604*	*0.856*		*0.672*	*0.742*	0.463	*0.594*	*− 0.891*
Leachate	(°C)	*0.840*	*0.744*	*0.672*		*0.863*	*0.660*	*0.617*	− 0.574
Amount leachate	(m^3^)	*0.876*	*0.714*	*0.742*	*0.863*		*0.723*	0.536	*− 0.667*
Air temperature	(°C)	*0.941*	0.407	0.463	*0.660*	*0.723*		0.390	− 0.468
Precipitation	(mm)	0.507	*0.770*	*0.594*	*0.617*	0.536	0.390		− 0.571
Waste	(Mg)	− 0.545	*− 0.764*	*− 0.891*	− 0.574	*− 0.667*	− 0.468	− 0.571	
Air temperature	(°C)	0.085	0.342	0.061	0.141	*0.723*		0.390	− 0.468
Precipitation	(mm)	− 0.277	0.360	0.017	0.521	0.536	0.390	1.000	− 0.571
Waste	(Mg)	− 0.082	− 0.477	0.177	− 0.545	*− 0.667*	− 0.468	− 0.571	
W2	(mg O_2_ L^−1^)		*0.645*	0.388	0.569	*− 0.843*	− 0.503	− 0.244	0.414
P5	(mg O_2_ L^−1^)	*0.645*		0.299	0.475	*− 0.659*	− 0.492	− 0.234	*0.648*
P6	(mg O_2_ L^−1^)	0.388	0.299		*0.591*	− 0.270	− 0.522	0.061	0.184
Leachate	(mg O_2_ L^−1^)	0.569	0.475	*0.591*		*− 0.707*	*− 0.612*	− 0.227	0.503
Amount leachate	(m^3^)	*− 0.843*	*− 0.659*	− 0.270	*− 0.707*		*0.723*	0.536	*− 0.667*
Air temperature	(°C)	− 0.503	− 0.492	− 0.522	*− 0.612*	*0.723*		0.390	− 0.468
Precipitation	(mm)	− 0.244	− 0.234	0.061	− 0.227	0.536	0.390		− 0.571
Waste	(Mg)	0.414	0.648	0.184	0.503	*− 0.667*	− 0.468	− 0.571	
W2	(mg NH4-N L^−1^)		0.096	*0.749*	− 0.133	0.178	0.375	0.042	− 0.303
P5	(mg NH4-N L^−1^)	0.096		− 0.112	− 0.108	0.332	0.349	− 0.423	0.105
P6	(mg NH4-N L^−1^)	*0.749*	− 0.112		0.268	0.143	0.171	0.138	− 0.468
Leachate	(mg NH4-N L^−1^)	− 0.133	− 0.108	0.268		0.002	− 0.262	− 0.129	− 0.178
Amount leachate	(m^3^)	0.178	0.332	0.143	0.002		*0.723*	0.536	*− 0.667*
Air temperature	(°C)	0.375	0.349	0.171	− 0.262	*0.723*		0.390	− 0.468
Precipitation	(mm)	0.042	− 0.423	0.138	− 0.129	0.536	0.390		− 0.571
Waste	(Mg)	− 0.303	0.105	− 0.468	− 0.178	*− 0.667*	− 0.468	− 0.571	

Italicizing the value of statistics means that the relationship is statistically significant at *p* < 0.05

The total amount of waste deposited at the landfill in the years 2009 to 2018 amounted to 8.033 Mg with 1.396 Mg of waste deposited within 12 months. Overall, the amount of waste deposited per day did not exceed 10 Mg. At the landfill, a downward trend in the amount of waste accepted for depositing was noted.

## Discussion

One of the most serious risks in landfills is the presence of leachate. The highest amount of leachate in the landfill occurred in summer, and the highest precipitation of 173.5 mm occurred in autumn with an overall average of 66.18 mm. The higher precipitation in the surroundings of the landfill in the Ghana area was shown by Boateng and Opoku (2019) at the level of 214.3 mm. The magnitude of these parameters over the course of 12 months was characterised by a downward trend. Öman (2008) suggested that the change in the physicochemical properties of the leachate may depend not only on the amount of leachate but also on the climatic conditions. Similarly, Foo and Hameed (2009) showed that leachate composition is also determined by the climate.

The deterioration of surface water quality in the landfill area was affected only by the COD concentration, the average outflow result of which differed slightly by 1.25 mg L^−1^ in relation to the inflow test of this determination. The analysis of groundwater quality in piezometers located in the landfill area showed that most of the examined physicochemical elements, including the heavy metal content, meet very good water quality standards at class I. Concentrations of chromium, cadmium, and mercury and other metals in leachate at a pH close to neutral (7.5) remained at a low level. According to Naveen et al. (2017), the low concentrations of heavy metals in the examined leachate at the highest average for zinc 0.12 mg L^−1^ confirms that the trace heavy metal concentration only indicated that the dumped waste was predominantly municipal waste. In the opinion of Kanownik and Policht-Latawiec (2016), such a state is characteristic of old landfills. In addition, the neutral reaction in the tested waters in the area of the landfill was also demonstrated by Kapelewska et al. (2016). The reaction of the tested leachate was also close to neutral. The relatively normal band of pH values in the leachate has also been shown by other researchers (Naveen et al. 2017). However, Brennan et al. (2016) observed a lower pH level in other landfills. According to some researchers (Nanny and Ratasuk 2002), this may be due to the age of the landfill, as high contents of metals and organic compounds are usually observed in newer landfills (Tatsi et al. 2003).

Below the landfill, the quality of groundwater practically deteriorated as a result of the average total nitrogen concentration exceeding 20 mg L^−1^. The source of such pollution is landfill sites (Almasri and Kaluarachchi 2004). Deterioration of water classification was also supported by non-class groundwater quality at the outflow in the landfill area as a result of exceeding the limit value by the mean concentration of the determined PAHs (0.008 mg L^−1^). An average lower than 0.003 mg L^−1^ was only found in inflow waters of unsatisfactory quality. The PAHs originate mainly from anthropogenic processes, in particular from incomplete combustion of organic fuels, and are widely distributed in the environment (Malakahmada et al. 2016). Some researchers have shown that organic compounds can easily penetrate water or soil if the isolation between deposited waste and soil is insufficient (Kapelewska et al. 2016; Nomngongo et al. 2012). The degree of contamination of aquifers depends on the speed of transport and storage conditions at the place where they penetrate the soil structure (Vasanthi et al. 2008). Differently selected physicochemical elements of surface water (temperature, pH, EC, DO, and NH_4_-N) did not show deterioration of the quality class of the water.

Another rarely used factor is the ambient temperature to identify the influence on the course of processes in the waste deposit. The average value of atmospheric air temperature in the landfill area close to 12 °C is considered by some researchers to be a factor that may influence the processes of organic matter degradation, ammonification, nitrification, and denitrification in leachate (Platzer 1996; Kadlec and Knight 1996). Groundwater was another place to study the temperature in the landfill site. In this case, the results of measurements at the four piezometric points confirmed that the acceptable level of purity at class I was exceeded at the highest mean of 11.35 °C at the inflow. Similarly, average EC values in the piezometers tested confirmed the level exceeding class I cleanliness due to reaching the highest average EC of 840 μS cm^−1^ at the point below the landfill. Alslaibi et al. (2011) found significantly higher values of EC between 1060 and 2350 μS cm^−1^ in the groundwater around the landfill. In turn, the highest mean concentration of NH_4_-N occurred at the inflow of the water, which resulted in the classification as class II. In general, transported pollutants contained in the groundwater with a much slower flow than the surface water may enter the latter through the inflow. In general, groundwater advection to the surface water is low, but the concentration of pollution increases when the surface water flows in as a result of percolation through sediment accumulation under the influence of sedimentation (Förstner and Wittmann 1979). At the same time, the pollution load increases. The presence of one of these processes is indicated by an increased content of NO_3_-N and PAHs. The DO content has statistically significant differences, indicating its decrease in groundwater at the outflow in the examined landfill area with an average content of 0.05 and 0.22 mg L^−1^. A significant difference occurred between the content of dissolved oxygen at *p* < 0.05, which showed a reduction of this determination in the examined waters below the analysed landfill. A lower average DO content in the water near the landfill was demonstrated by Gamar et al. (2018). Other researchers (Diaz 2001; Kronvang et al. 2005) have suggested that the presence of dissolved organic carbon and ammonium nitrogen could be a factor in reducing DO content in water (Guo et al. 2010).

On the basis of the correlation matrix analysis, it was proved that the old landfill has a certain effect on surface water and groundwater pollution, despite not exceeding the permissible content at the highest average concentration of NH_4_-N at 1.34 mg L^−1^. Higher content of NH_4_-N in groundwater around of the landfill have been shown by Preziosi et al. (2019). The concentration of this indicator in groundwater below the landfill in one piezometer was positively correlated with the depth of water retention. Regarding such an increase in pollution along with the depth of groundwater, Wu et al. (2016) justified the permeation of N with increased atmospheric waste. Christensen et al. (2000) and Thomsen et al. (2012) classified ammonium nitrate as one of the main factors of water pollution in the landfill area on the basis of a mass load of pollutants. This confirms that the spatial scope of groundwater risk through the source of contamination (i.e. the landfill) is inevitably related to the permeation of the liquid phase and to the type and amount of leachate (Przydatek 2012).

Examination of water within the old landfill indicates an interaction with leachate waters, which was also suggested by Przydatek and Kanownik (2019) and by Nanny and Ratasuk (2002). This may be due to the insufficient performance of the existing drainage system after a period of 19 years of use. Bashir et al. (2009) identified landfills as one of the main sources of surface water and groundwater pollution, where, if not properly addressed, leachate can penetrate the soil and reach aquifers. However, Thomsen et al. (2012) included surface runoffs or improper handling of the landfill as factors affecting surface water.

The results showed that the landfill under investigation, due to more than 10 years of operation, should be classified as a stabilised landfill (Kjeldsen et al. 2002). However, Han et al. (2016) demonstrated that the most intensive groundwater pollution occurs in the area of landfills not exceeding 20 years of age. Demonstrated contaminants migrating from the landfill may occur in the immediate vicinity, even at a distance of up to several hundred metres. The location of the landfill itself may contribute to the spread of pollution from the landfill to the surrounding aquatic environment. The range of their effect usually depends on local geological and hydrogeological conditions as well as dilution processes, redox reaction, ion exchange in the ground, and the water environment (Alslaibi et al. 2011; Banu and Berrin 2015).

## Conclusions

On the basis of a multi-disciplinary analysis of the quality of the water environment in the old landfill area, including its useful life, the following conclusions can be drawn:

- The quality of water within the landfill below its location showed changes in the classification of the quality, suggesting a moderate influence of the old landfill.
- The content of PAHs in groundwater was increased, and a noticeable increase in the concentration of organic compounds in relation to the result in incoming waters resulted in the classification of the groundwater as a non-class quality, with an indication of the possibility of landfill influence.
- Significantly lower DO content in groundwater below the landfill may have been caused by anthropogenic factors.
- The statistically significant relation between the content of ammoniacal nitrogen in the surface water and groundwater below the landfill and the increase in the concentration of NH_4_-N with the depth of groundwater indicates the possibility of water seeping into this environment.
- The interaction of leachate water on surface and groundwater in the old landfill area indicates the possibility of uncontrolled surface runoff or limited efficiency of the leachate water intake system.
- The list of legally required parameters of indicators used for monitoring water in landfill areas should be extended to include determinations for NH_4_-N and DO.
